# Prediction and visualization map for physicochemical indices of kiwifruits by hyperspectral imaging

**DOI:** 10.3389/fnut.2024.1364274

**Published:** 2024-03-14

**Authors:** Qinglong Meng, Tao Tan, Shunan Feng, Qingchun Wen, Jing Shang

**Affiliations:** ^1^School of Food Science and Engineering, Guiyang University, Guiyang, China; ^2^Research Center of Nondestructive Testing for Agricultural Products of Guizhou Province, Guiyang, China

**Keywords:** kiwifruits, physicochemical index, visualization, nondestructive detection, hyperspectral imaging, chemometrics

## Abstract

Soluble solid content (SSC), firmness, and color (L^*^, a^*^, and b^*^) are important physicochemical indices for assessing the quality and maturity of kiwifruits. Therefore, this research aimed to realize the nondestructive detection and visualization map for the physicochemical indices of kiwifruits at different maturity stages by hyperspectral imaging coupled with the chemometrics. To further improve the detection accuracy and working efficiency of the models, competitive adaptive reweighted sampling (CARS) and successive projection algorithm were employed to choose feature wavelengths for predicting the physicochemical indices of kiwifruits. Multiple linear regression (MLR) was designed to develop simplified detection models based on feature wavelengths for determining the physicochemical indices of kiwifruits. The results showed that 32, 18, 26, 29, and 32 feature wavelengths were extracted from 256 full wavelengths to predict the SSC, firmness, L^*^, a^*^, and b^*^, respectively, with the CARS algorithm. Not only was the working efficiency of the CARS-MLR model improved, but the prediction accuracy of the CARS-MLR model for determining the physicochemical indices was also at its relative best. The residual predictive deviations of the CARS-MLR model for determining the SSC, firmness, L^*^, a^*^, and b^*^ were 3.09, 2.90, 2.32, 2.74, and 2.91, respectively, which were all above 2.3. Compared with the model based on the full spectra, the CARS-MLR model could be used to predict the physicochemical indices of kiwifruits. Finally, the visualization map for the physicochemical indices of kiwifruits at different maturity stages was generated by calculating the spectral response of each pixel on the kiwifruit samples with the CARS-MLR model. This made the detection for the physicochemical indices of kiwifruits more intuitive. This study demonstrates that hyperspectral imaging coupled with the chemometrics is promising for the nondestructive detection and visualization map for the physicochemical indices of kiwifruits, and also provides a novel theoretical basis for the nondestructive detection of kiwifruit quality.

## 1 Introduction

As one of the post-ripening fruits, kiwifruit is usually picked unripe during actual harvesting to prolong its storage time. If picked too early, kiwifruits will retain their flesh firmness, thereby affecting the taste. Harvesting too late will lead to over-ripeness, making it difficult to store kiwifruits (1). Soluble solid content (SSC), firmness, and color (L^*^, a^*^, and b^*^) are essential parameters for assessing kiwifruit quality and ripeness. Therefore, nondestructive detection of the SSC, firmness, and color of kiwifruits at different maturity stages can be useful for determining the appropriate harvesting time and post-harvest quality grading.

Although traditional methods for measuring the physicochemical indices of fruits are characterized by relatively high accuracy, they have their fair share of disadvantages. For example, they may destroy the detected objects, the detection is inefficient and time-consuming, and large-scale detection is difficult to realize (2, 3). Nondestructive detection methods based on hyperspectral imaging have been widely used in the field of fruit quality detection (4–9), which are fast and non-contaminating compared with traditional detection methods. The hyperspectral imaging technique can not only quickly and nondestructively capture the spectral information of the object of interest but also obtain its image information (10). By integrating spectral and image information, this technique can rapidly and nondestructively detect the internal and external quality of fruits. Compared with near-infrared spectroscopy, hyperspectral imaging can obtain the spectral information of the entire object of interest, which helps construct a more stable prediction model (11). In addition, the image information obtained by hyperspectral imaging can be used to visualize the distribution of the physicochemical indices of fruits, making the detection of these indices more intuitive. However, hyperspectral images are characterized as high dimensionality data, which will influence the detection efficiency. Fan et al. (12) employed hyperspectral imaging technology to quantitatively predict SSC and firmness in Korla pears. Partial least square regression (PLSR) models based on variables extracted from the combination of competitive adaptive reweighted sampling (CARS) and successive projection algorithm (SPA) demonstrated outstanding prediction performance. The correlation coefficients and root mean square errors of the prediction set (RMSEP) were 0.88 and 0.49 for SSC, and 0.87 and 0.72 for firmness. Xie et al. (13) proposed a method of using hyperspectral imaging to quantitatively predict the color (L^*^, a^*^, and b^*^) and firmness of bananas. The constructed partial least squares prediction model performed relatively well. The coefficient of determination in the prediction set was 0.80 for L^*^, 0.97 for a^*^, 0.77 for b^*^, and 0.76 for firmness. Su et al. (14) combined hyperspectral imaging and deep learning to quantitatively predict strawberry SSC and identify strawberry ripeness. Wang et al. (15) investigated the feasibility of evaluating the apple quality and ripeness using hyperspectral imaging; the results showed that the constructed model could achieve nondestructive detection and spatial distribution of apple quality and ripeness. No study has been reported on quantitative prediction and distribution visualization of multiple physicochemical indices of kiwifruits using hyperspectral imaging.

Therefore, this study constructed a model for predicting the physicochemical indices of kiwifruits at different maturity stages based on hyperspectral imaging and chemometrics. To improve the detection accuracy and efficiency of the prediction model, we used CARS and SPA algorithms to select feature wavelengths for the physicochemical indices of kiwifruits, to construct a simplified prediction model. The distributions map of the physicochemical indices of kiwifruits at different maturity stages were visualized using the pseudo-color technique, making the detection of such indices more intuitive. This study provides a reference for the accurate prediction of the physicochemical indices of kiwifruits in actual production.

## 2 Materials and methods

### 2.1 Kiwifruit samples

The “Guichang” kiwifruit samples used in this study were obtained from the commercial orchard in Xiuwen County, Guizhou Province, China. To improve the detection range of the physicochemical indices of kiwifruits, we randomly picked samples from kiwifruit trees at different locations in four batches from September to October 2019. Samples were picked at different maturity stages (unripe stage: picked on September 17, with the SSC ranging from 4.63 to 5.73 °Brix; semi-ripe stage: picked on September 28, with the SSC ranging from 5.90 to 7.30 °Brix; ripe stage: picked on October 7, with the SSC ranging from 6.50 to 9.20 °Brix; over-ripe stage: picked on October 19, with the SSC ranging from 9.20 to 12.20 °Brix). Fifty samples free of defects and damages were selected from each batch, totalling 200 samples. Before the experiment, the dust on the surface of the samples was wiped off with soft paper. The samples were sequentially numbered to obtain the hyperspectral images and measure the physicochemical indices.

### 2.2 Instruments and equipment

The following instruments and equipment were used: GaiaField-F-V10 Hyperspectral Imaging System (Jiangsu Dualix Spectral Imaging Technology Co., Ltd.); ATAGO PAL-*α* Refractometer (Atago, Japan); TD4Z-WS Centrifuge (Hunan Cenlee Scientific Instruments Co., Ltd.); GY-4 Sclerometer (Hangzhou Lvbo Instrument Co., Ltd.); Ci7800 Spectrophotometer [X-Rite (Shanghai) Color Instrument Trading Co. Ltd.].

**Figure 1:**
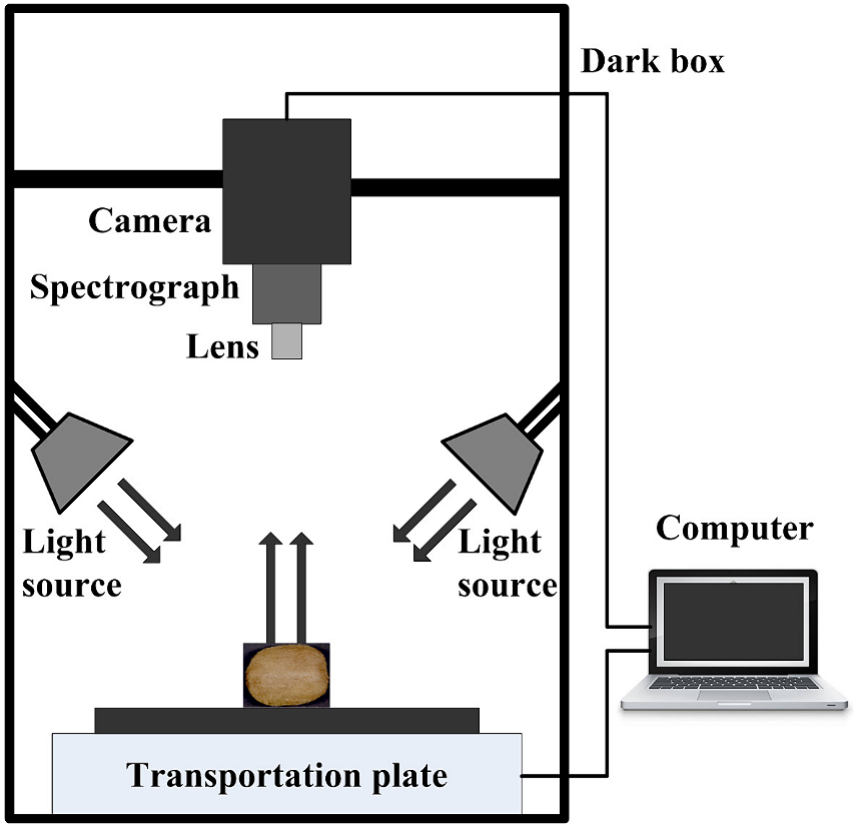
Configuration diagram of hyperspectral imaging system.

Figure 1 shows the configuration diagram of the hyperspectral imaging system, of which the wavelength ranges from 390 nm to 1,030 nm. The spatial resolution of hyperspectral images is 0.2 mm/pixels. The parameters of the instrument are detailed in the reference (16).

### 2.3 Hyperspectral images acquisition and correction

We placed the numbered kiwifruit samples on an electric transportation plate and scanned them one by one to obtain their original hyperspectral images. Due to the uneven intensity distribution of the light source in the dark box and certain differences in sample shapes, there were noise signals in the acquired original hyperspectral images. After acquiring the hyperspectral images of all the samples, we acquired black-and-white calibrated images with the same system parameters, and performed reflectance correction for the original hyperspectral images of all the samples as follows (formula 1):

(1) $$R_{ref}=\frac{R_{\mathrm{original}}-R_{\mathrm{black}}}{R_{\mathrm{white}}-R_{\mathrm{black}}}$$

where *R*_ref_ is the calibrated image, *R*_original_ is the original image, and *R*_white_ and *R*_black_ are the white and black calibrated images, respectively.

### 2.4 Measurement of physicochemical indices

After acquiring the hyperspectral images of all the samples, we measured the color values (L^*^, a^*^, and b^*^) of kiwifruits at the equatorial positions using a spectrophotometer. The aperture plate diameter of the spectrophotometer was 10 mm. After the spectrophotometer was calibrated, we placed the samples in the observer of the spectrophotometer, and then gently closed the color sample holder to determine the color values.

The reference firmness of the samples was determined using a sclerometer. The probe diameter of the sclerometer was 7.9 mm, and the depth at which the probe was pressed into the kiwifruit flesh was roughly 10 mm. The skin of the samples near the equator was peeled before the measurement. The probe was aligned with the flesh of the fruit and then pressed slowly and uniformly to the mark. Each sample was tested thrice. The average of the tested values was used as the reference firmness of the samples.

The reference SSC of the kiwifruit samples was determined by a refractometer with a range of 0.0–85 °Brix and an accuracy of ±0.2 °Brix. Juice was extracted from the samples and then centrifuged at 3000 r/min for 5 min before the determination. The sample juice was smeared on a mirror, followed by multiple consecutive measurements until the last three measurements were the same. Afterwards, this measurement was taken as the reference SSC of the samples.

### 2.5 Modeling and assessment

A PLSR detection model was constructed to predict the physicochemical indices of kiwifruits based on the full-band data and the reference values of the indices. CARS (17) and SPA (18) were used to select feature wavelengths. SPA adopts an easy projection procedure in a vector space to select subsets of wavelengths with minimum colinearity. CARS employs the absolute values of regression coefficients of PLSR model as a parameter for evaluating the importance of every wavelength. Based on the selected characteristic spectra and the reference values, we then constructed a simplified multiple linear regression (MLR) model to predict the physicochemical indices of kiwifruits. The linear regression equation between the independent variable *X* (*X*_1_, *X*_2_, *X*_3_,.., *X_m_*) and the dependent variable *Y* (each physicochemical index) is given as follows (formula 2):

(2) $$Y=\beta_{0}+\beta_{1}X_{1}+\ldots +\beta_{m}X_{m}+\varepsilon$$

Assuming that the observations are {(*Y_i_*, *X*_*i*1_,.., *X_im_*), *i* = 1, 2,.., *n*}, the MLR prediction model is as follows (formula 3):

(3) $$\begin{array}{l} Y_{1}=\beta_{0}+\beta_{1}X_{11}+\beta_{2}X_{12}+\ldots +\beta_{m}X_{1m}+\varepsilon_{1}\\ Y_{2}=\beta_{0}+\beta_{1}X_{21}+\beta_{2}X_{22}+\ldots +\beta_{m}X_{2m}+\varepsilon_{2}\\ \ldots \\ Y_{n}=\beta_{0}+\beta_{1}X_{n1}+\beta_{2}X_{n2}+\ldots +\beta_{m}X_{\mathrm{nm}}+\varepsilon_{n} \end{array}\}$$

where *β_i_* denotes the coefficient of the *i*th independent variable, and *ε_i_* denotes the error term.

The coefficient of determination (*R*^2^_C_) and its root mean square error (RMSEC) for the calibration set, the coefficient of determination (*R*^2^_P_) and its root mean square error (RMSEP) for the prediction set, and the residual prediction deviation (RPD) were used as the key indicators to determine the prediction model performance (formulas 4–8). The prediction model is more effective if *R*^2^_C_ and *R*^2^_P_ are closer to 1, RMSEC and RMSEP are closer to 0, and the RPD value is larger. For the model, 1.4 ≤ RPD < 1.8 indicates a poor prediction effect; 1.8 ≤ RPD < 2.0 indicates a good prediction effect; RPD ≥ 2.0 indicates an excellent prediction effect (19).

(4) $$R_{C}^{2}=1-\frac{\overset{n_{C}}{\underset{i=1}{\sum }}{\left[y_{\mathrm{act}}\left(i\right)-y_{\mathrm{cal}}\left(i\right)\right]}^{2}}{\overset{n_{C}}{\underset{i=1}{\sum }}{\left[y_{\mathrm{act}}\left(i\right)-y_{\mathrm{mean}}\left(i\right)\right]}^{2}}$$

(5) $$R_{P}^{2}=1-\frac{\overset{n_{P}}{\underset{i=1}{\sum }}{\left[y_{\mathrm{act}}\left(i\right)-y_{pre}\left(i\right)\right]}^{2}}{\overset{n_{P}}{\underset{i=1}{\sum }}{\left[y_{\mathrm{act}}\left(i\right)-y_{\mathrm{mean}}\left(i\right)\right]}^{2}}$$

(6) $$\mathrm{RMSEC}=\sqrt{\frac{1}{n_{C}}\overset{n_{C}}{\underset{i=1}{\sum }}{\left[y_{\mathrm{act}}\left(i\right)-y_{\mathrm{cal}}\left(i\right)\right]}^{2}}$$

(7) $$\mathrm{RMSEP}=\sqrt{\frac{1}{n_{P}}\overset{n_{P}}{\underset{i=1}{\sum }}{\left[y_{\mathrm{act}}\left(i\right)-y_{pre}\left(i\right)\right]}^{2}}$$

(8) $$RPD=\frac{SD}{\mathrm{RMSEP}}$$

where *n_C_* and *n_P_* are the numbers of samples in the calibration and prediction set, respectively; *y*_act_ and *y*_mean_ are the reference and mean values of the physicochemical indices, respectively; *y*_cal_ and *y*_pre_ are the predicted values of the physicochemical indices in the calibration and prediction set, respectively; SD is the standard deviation of the reference values of the physicochemical indices in the prediction set.

## 3 Results and discussion

### 3.1 Statistics of the physicochemical indices

**Figure 2:**
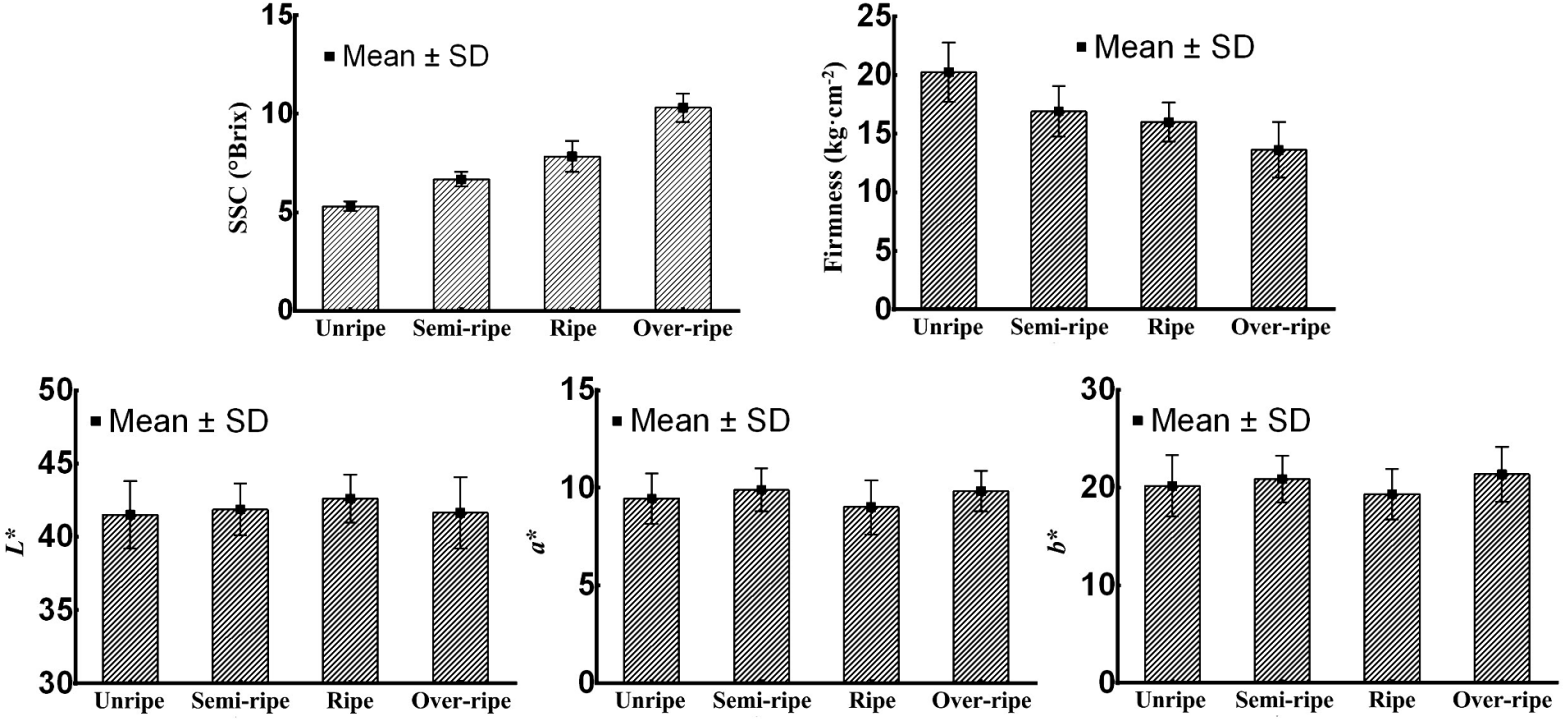
Physicochemical indices of kiwifruit samples at different maturity stages.

Figure 2 shows the statistical results for the physicochemical indices of kiwifruits at different maturity stages. It can be seen that the mean SSC of kiwifruits at different maturity stages increased with their gradual maturity, while the mean firmness of kiwifruits at different maturity stages showed the opposite trend of change. The color values of kiwifruits at different maturity stages changed relatively little, among which a^*^ and b^*^ followed the same trend.

Before a model for predicting the physicochemical indices of kiwifruits is constructed, all the samples need to be divided into calibration and prediction sets based on the acquired reflectance spectral data and measured physicochemical values. Whether the ranges of physicochemical values in the calibration set are larger than those in the prediction set is usually taken as the basis for evaluating the advantages and disadvantages of dividing the sample sets. The joint *x*-*y* distance method based on spectral data and physicochemical values (SPXY) (20) was used to divide 200 kiwifruit samples into 140 calibration sets and 60 prediction sets. The statistics of the sample sets divided using the SPXY algorithm are presented in Table 1. It can be seen that the value ranges of all physicochemical indices in the calibration set were wider than those in the prediction set.

**Table 1:** Statistics of partitioning sample sets using the SPXY algorithm.

Number of samples	Index	Min	Max	Mean ± SD
Calibration set (140)	SSC (°Brix)	4.80	11.50	8.13 ± 1.90
	Firmness (kg·cm^−2^)	12.01	24.01	18.07 ± 3.38
	*L* ^*^	37.04	45.92	41.62 ± 2.50
	*a* ^*^	6.82	11.57	9.21 ± 1.30
	*b* ^*^	15.23	25.44	20.12 ± 2.83
Prediction set (60)	SSC (°Brix)	5.10	11.40	8.02 ± 1.95
	Firmness (kg·cm^−2^)	12.34	23.98	17.20 ± 3.16
	*L* ^*^	37.59	45.33	41.58 ± 2.25
	*a* ^*^	6.88	11.52	9.66 ± 1.29
	*b* ^*^	15.71	25.43	20.76 ± 2.82

### 3.2 Spectral preprocessing

**Figure 3:**
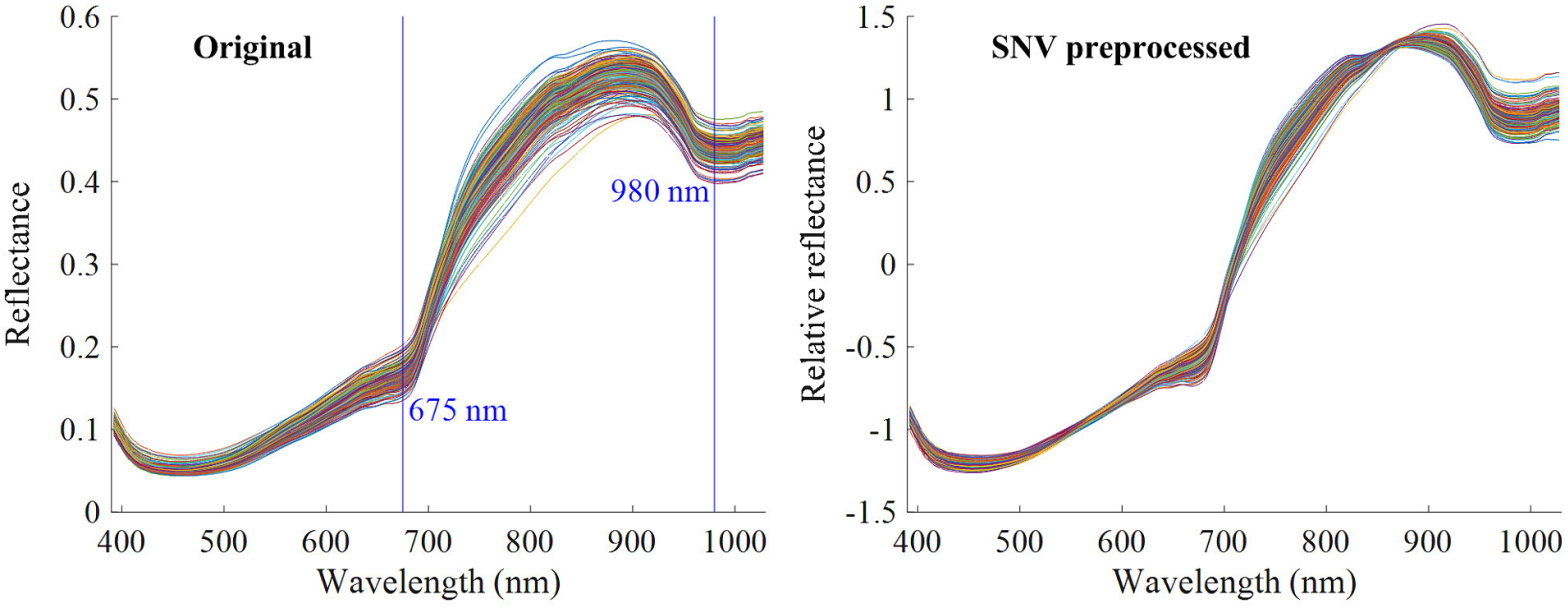
Reflectance spectra of kiwifruits.

Due to the influence of the particle size and surface scattering of the samples (21, 22), some noise is included in the acquired original spectra. To improve the prediction accuracy and stability of the detection model, this study used standard normal variation (SNV) to eliminate the noise in the original spectra (16). Figure 3 presents the original spectra of all the samples and the relative reflectance spectra after SNV preprocessing. The selected region of interest (ROI) is the entire sample. As can be seen in Figure 3, the waveforms of all spectral curves exhibited the same trend, with a more pronounced absorption peak near the 675 nm wavelength. This is possibly caused by chlorophyll absorption. Another absorption peak occurred near 980 nm, which may be related to the absorption of water in kiwifruits. Comparing Figure 3, we can observe that the relative reflectance spectra after SNV preprocessing were smoother than the original spectra, indicating that SNV preprocessing can eliminate part of the noise in the original spectra.

### 3.3 Feature wavelength extraction

Two techniques, SPA and CARS, were used to extract feature wavelengths to improve the computational efficiency of the model. By comparing these two techniques, we selected the better feature wavelength extraction method for the prediction of the physicochemical indices of kiwifruits.

#### 3.3.1 Extraction of feature wavelengths by SPA

When the SPA method was used to select feature wavelengths, the number of effective feature wavelengths was usually determined based on the lowest root mean squared error (RMSE) of the model. Firstly, the RMSE value decreased with the increase in the number of effective wavelengths. Then, when the number of effective wavelengths in the model increased to a certain value, the decreasing tendency of the RMSE value was no longer evident. In this study, the RMSE values no longer decreased when the number of effective wavelengths was greater than 10, 8, 13, 9, and 14 for the prediction of SSC, firmness, L^*^, a^*^, and b^*^, respectively. Therefore, we extracted 10, 8, 13, 9, and 14 wavelengths as the feature wavelengths for predicting SSC, firmness, L^*^, a^*^, and b^*^. The proportion of the feature wavelengths by SPA in the total of 256 wavelengths is 3.91, 3.13, 5.08, 3.52, and 5.47%, respectively. The selected feature wavelengths are presented in Table 2.

**Table 2:** Feature wavelengths for predicting the physicochemical indices of kiwifruits selected by SPA and CARS.

Index	Methods	Band Num	Wavelength (nm)
SSC	CARS	32	393, 400, 407, 428, 430, 432, 439, 446, 453, 460, 600, 614, 878, 883, 891, 899, 904, 907, 920, 922, 925, 938, 941, 946, 948, 956, 959, 972, 988, 993, 996, 1012
	SPA	10	428, 432, 439, 446, 458, 580, 614, 656, 1017, 1025
Firmness	CARS	18	397, 402, 421, 432, 556, 590, 626, 824, 889, 904, 946, 954, 964, 972, 975, 985, 1009, 1012
	SPA	8	393, 397, 400, 402, 404, 409, 411, 442
*L* ^*^	CARS	26	400, 402, 404, 428, 522, 592, 595, 597, 626, 629, 631, 634, 636, 639, 698, 715, 721, 814, 824, 928, 1006, 1009, 1017, 1020, 1022, 1028
	SPA	13	393, 400, 402, 407, 411, 484, 525, 573, 653, 696, 711, 907, 998
*a* ^*^	CARS	29	402, 423, 430, 435, 453, 458, 489, 597, 617, 624, 626, 666, 683, 746, 789, 791, 794, 822, 827, 855, 904, 920, 956, 975, 977, 983, 998, 1001, 1004
	SPA	9	407, 458, 520, 556, 678, 706, 852, 876, 964
*b* ^*^	CARS	32	411, 416, 418, 423, 430, 439, 449, 463, 520, 551, 553, 592, 595, 597, 617, 619, 622, 624, 626, 646, 746, 768, 786, 804, 806, 922, 951, 969, 988, 996, 1020, 1022
	SPA	14	411, 421, 425, 449, 522, 549, 590, 619, 863, 870, 938, 964, 1017, 1028

#### 3.3.2 Extraction of feature wavelengths by CARS

When the CARS method was used to select feature wavelengths, the number of Monte Carlo sampling runs and the group amount for cross-validation were 50 and 5. For predicting SSC, firmness, L^*^, a^*^, and b^*^, the root mean square error of cross validation (RMSECV) at different sampling runs exhibited a consistent trend, i.e., first decreasing and then increasing. The smallest RMSECV was obtained at the 22nd, 28th, 24th, 23rd, and 22nd sampling runs, respectively. This optimal wavelength subset contained 32, 18, 26, 29, and 32 feature wavelengths for the prediction of SSC, firmness, L^*^, a^*^, and b^*^. The proportion of the feature wavelengths by CARS in the total of 256 wavelengths is 12.50, 7.03, 10.16, 11.33, and 12.50%, respectively. The selected feature wavelengths are given in Table 2.

### 3.4 Modeling results

**Figure 4:**
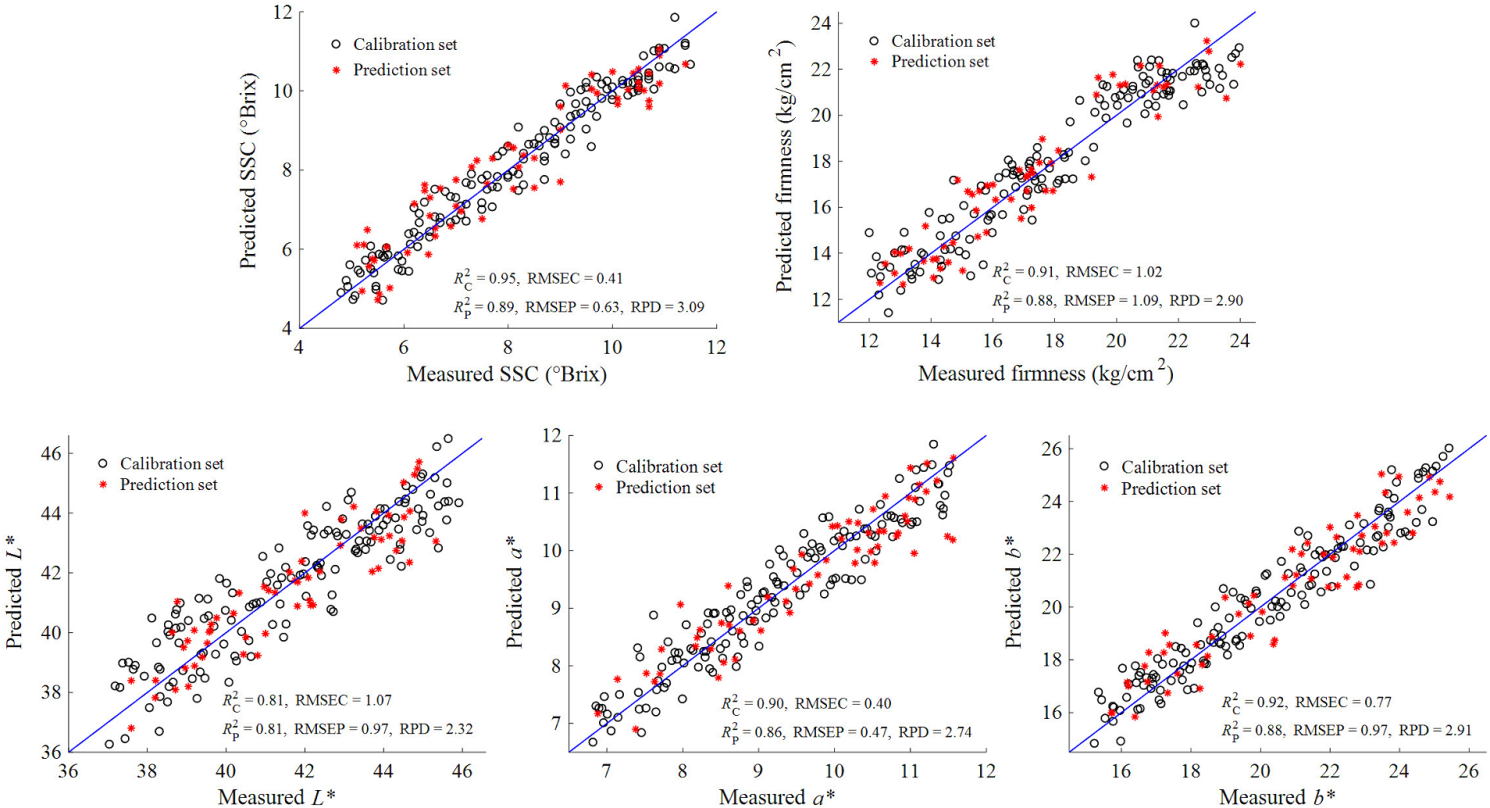
Prediction results for physicochemical indices of kiwifruits by CARS-MLR model.

In this study, we first constructed a PLSR model for predicting the physicochemical indices of kiwifruits based on 256 full-band variables and measured reference values. Then, we established a simplified MLR model for predicting the physicochemical indices of kiwifruits by taking the feature wavelengths selected by SPA and CARS as independent variables and the reference values of such physicochemical indices as dependent variables. The prediction results for the physicochemical indices of kiwifruits by PLSR and MLR models are listed in Table 3. These results indicate that, for the prediction of each physicochemical index of kiwifruits, the *R*^2^_C_, *R*^2^_P_, and RPD values of the CARS-MLR model were higher than those of the SPA-MLR model, and the RMSEC and RMSEP of the CARS-MLR model were lower than those of the SPA-MLR model. This indicates that the CARS algorithm was a better feature wavelength selection method compared to the SPA algorithm. By comparing the PLSR model based on full wavelengths and the CARS-MLR model based on feature wavelengths in Table 3, we can see that for the prediction of each physicochemical index of kiwifruits, the *R*^2^_C_, *R*^2^_P_, and RPD values of the latter model were higher than those of the former model. Moreover, the RMSEC and RMSEP of the CARS-MLR model were also lower than those of the PLSR model, indicating an improvement in both the operational efficiency and detection performance of the CARS-MLR model. In addition, the RPD values of the CARS-MLR model for predicting SSC, firmness, L^*^, a^*^, and b^*^ were 3.09, 2.90, 2.32, 2.74, and 2.91, respectively. These values were all greater than 2.0, further indicating the excellent prediction effect of the CARS-MLR model. The results of the CARS-MLR model for each physicochemical index of kiwifruits are given in Figure 4. It can be seen from Figure 4 that the linear relationship between the predicted and measured values of the physicochemical index were relatively favorable for all samples except for a few deviating from the regression line, indicating that the CARS-MLR model can accurately predict the physicochemical index of kiwifruits. The CARS-MLR model formulas (9–13) for predicting each physicochemical index of kiwifruits are given as follows:

(9) $$\begin{array}{c} Y_{SSC}=-142.2-62.4X_{393\mathrm{nm}}+111.4X_{400\mathrm{nm}}-75.9X_{407\mathrm{nm}}\\ +146.5X_{428\mathrm{nm}}+75.9X_{430\mathrm{nm}}\\ -214.4X_{432\mathrm{nm}}+179.3X_{439\mathrm{nm}}\\ -188.1X_{446\mathrm{nm}}-192.8X_{453\mathrm{nm}}+152.2X_{460\mathrm{nm}}\\ +42.4X_{600\mathrm{nm}}-99.5X_{614\mathrm{nm}}+57.8X_{878\mathrm{nm}}\\ +253.8X_{883\mathrm{nm}}-230.9X_{891\mathrm{nm}}-134.0X_{899\mathrm{nm}}\\ -64.9X_{904\mathrm{nm}}-186.6X_{907\mathrm{nm}}+169.5X_{920\mathrm{nm}}\\ -19.2X_{922\mathrm{nm}}+307.6X_{925\mathrm{nm}}-147.5X_{938\mathrm{nm}}\\ +227.0X_{941\mathrm{nm}}-265.4X_{946\mathrm{nm}}-205.0X_{948\mathrm{nm}}\\ +416.7X_{956\mathrm{nm}}+21.7X_{959\mathrm{nm}}-203.8X_{972\mathrm{nm}}\\ +96.1X_{988\mathrm{nm}}-70.4X_{993\mathrm{nm}}\\ -18.7X_{996\mathrm{nm}}+21.2X_{1012\mathrm{nm}} \end{array}$$

(10) $$\begin{array}{c} Y_{F\mathrm{irmness}}=235.6+208.4X_{397\mathrm{nm}}-197.2X_{402\mathrm{nm}}\\ -130.9X_{421\mathrm{nm}}+235.9X_{432\mathrm{nm}}+178.8X_{556\mathrm{nm}}\\ -234.1X_{590\mathrm{nm}}+203.0X_{626\mathrm{nm}}+51.2X_{824\mathrm{nm}}\\ -161.2X_{889\mathrm{nm}}+131.2X_{904\mathrm{nm}}+262.1X_{946\mathrm{nm}}\\ -362.4X_{954\mathrm{nm}}-279.4X_{964\mathrm{nm}}+345.4X_{972\mathrm{nm}}\\ +289.4X_{975\mathrm{nm}}-236.1X_{985\mathrm{nm}}\\ +290.5X_{1009\mathrm{nm}}-301.8X_{1012\mathrm{nm}} \end{array}$$

(11) $$\begin{array}{c} Y_{L^{*}}=197.6-85.7X_{400\mathrm{nm}}+108.5X_{402\mathrm{nm}}\\ +36.5X_{404\mathrm{nm}}-274.5X_{428\mathrm{nm}}-108.8X_{522\mathrm{nm}}\\ -254.2X_{592\mathrm{nm}}-1215.1X_{595\mathrm{nm}}+1922.1X_{597\mathrm{nm}}\\ -216.1X_{626\mathrm{nm}}-262.9X_{629\mathrm{nm}}+574.0X_{631\mathrm{nm}}\\ -2265.0X_{634\mathrm{nm}}+2142.0X_{636\mathrm{nm}}-620.6X_{639\mathrm{nm}}\\ +151.6X_{698\mathrm{nm}}-171.4X_{715\mathrm{nm}}-22.6X_{721\mathrm{nm}}\\ -143.8X_{814\mathrm{nm}}-58.6X_{824\mathrm{nm}}-190.2X_{928\mathrm{nm}}\\ -39.5X_{1006\mathrm{nm}}-95.0X_{1009\mathrm{nm}}-31.1X_{1017\mathrm{nm}}\\ -273.1X_{1020\mathrm{nm}}+184.8X_{1022\mathrm{nm}}+137.5X_{1028\mathrm{nm}} \end{array}$$

(12) $$\begin{array}{c} Y_{a^{*}}=54.9+40.7X_{402\mathrm{nm}}-73.1X_{423\mathrm{nm}}\\ -79.7X_{430\mathrm{nm}}+132.3X_{435\mathrm{nm}}-48.0X_{453\mathrm{nm}}\\ +192.8X_{458\mathrm{nm}}-114.3X_{489\mathrm{nm}}-290.3X_{597\mathrm{nm}}\\ +626.3X_{617\mathrm{nm}}-309.5X_{624\mathrm{nm}}-3.2X_{626\mathrm{nm}}\\ +77.1X_{666\mathrm{nm}}-71.6X_{683\mathrm{nm}}+48.9X_{746\mathrm{nm}}\\ +145.6X_{789\mathrm{nm}}-13.8X_{791\mathrm{nm}}-293.5X_{794\mathrm{nm}}\\ -14.8X_{822\mathrm{nm}}+245.2X_{827\mathrm{nm}}-132.5X_{855\mathrm{nm}}\\ +187.8X_{904\mathrm{nm}}-138.3X_{920\mathrm{nm}}-83.0X_{956\mathrm{nm}}\\ +36.8X_{975\mathrm{nm}}+92.8X_{977\mathrm{nm}}-29.6X_{983\mathrm{nm}}\\ +81.8X_{998\mathrm{nm}}+115.9X_{1001\mathrm{nm}}-214.9X_{1004\mathrm{nm}} \end{array}$$

(13) $$\begin{array}{c} Y_{b^{*}}=-29.1+60.6X_{411\mathrm{nm}}+90.9X_{416\mathrm{nm}}+130.2X_{418\mathrm{nm}}\\ -10.0X_{423\mathrm{nm}}-269.5X_{430\mathrm{nm}}-195.0X_{439\mathrm{nm}}\\ +294.3X_{449\mathrm{nm}}+61.7X_{463\mathrm{nm}}-191.4X_{520\mathrm{nm}}\\ +249.1X_{551\mathrm{nm}}-67.2X_{553\mathrm{nm}}+697.0X_{592\mathrm{nm}}\\ +207.6X_{595\mathrm{nm}}-1374.3X_{597\mathrm{nm}}-1011.1X_{617\mathrm{nm}}\\ +857.1X_{619\mathrm{nm}}+1120.6X_{622\mathrm{nm}}+754.0X_{624\mathrm{nm}}\\ -1003.2X_{626\mathrm{nm}}-150.5X_{646\mathrm{nm}}+191.1X_{746\mathrm{nm}}\\ -424.8X_{768\mathrm{nm}}+287.9X_{786\mathrm{nm}}+590.7X_{804\mathrm{nm}}\\ -526.7X_{806\mathrm{nm}}+135.2X_{922\mathrm{nm}}-132.0X_{951\mathrm{nm}}\\ +220.8X_{969\mathrm{nm}}-200.4X_{988\mathrm{nm}}+124.1X_{996\mathrm{nm}}\\ +83.2X_{1020\mathrm{nm}}-90.0X_{1022\mathrm{nm}} \end{array}$$

where *Y_SSC_*, *Y_Firmness_*, *Y*_L*_, *Y*_a*_, and *Y*_b*_ are the predicted values for each physicochemical index of kiwifruits. *X*_*i*nm_ represents the SNV preprocessed spectral value at the wavelength of *i* nm.

**Table 3:** Prediction results for the physicochemical indices of kiwifruits by the PLSR and MLR models.

Model	Index	Calibration set		Prediction set		
		*R*_C_^2^	RMSEC	*R*_P_^2^	RMSEP	RPD
PLSR	SSC	0.94	0.44	0.85	0.76	2.58
	Firmness	0.90	1.06	0.84	1.25	2.53
	*L* ^*^	0.79	1.15	0.79	1.03	2.18
	*a* ^*^	0.90	0.40	0.83	0.52	2.46
	*b* ^*^	0.91	0.86	0.88	0.98	2.88
SPA-MLR	SSC	0.75	0.94	0.88	0.68	2.89
	Firmness	0.87	1.23	0.81	1.38	2.29
	*L* ^*^	0.72	1.31	0.79	1.01	2.23
	*a* ^*^	0.78	0.61	0.81	0.55	2.34
	*b* ^*^	0.85	1.08	0.87	1.02	2.75
CARS-MLR	SSC	0.95	0.41	0.89	0.63	3.09
	Firmness	0.91	1.02	0.88	1.09	2.90
	*L* ^*^	0.81	1.07	0.81	0.97	2.32
	*a* ^*^	0.90	0.40	0.86	0.47	2.74
	*b* ^*^	0.92	0.77	0.88	0.97	2.91

### 3.5 Distribution visualization of physicochemical indices

**Figure 5:**
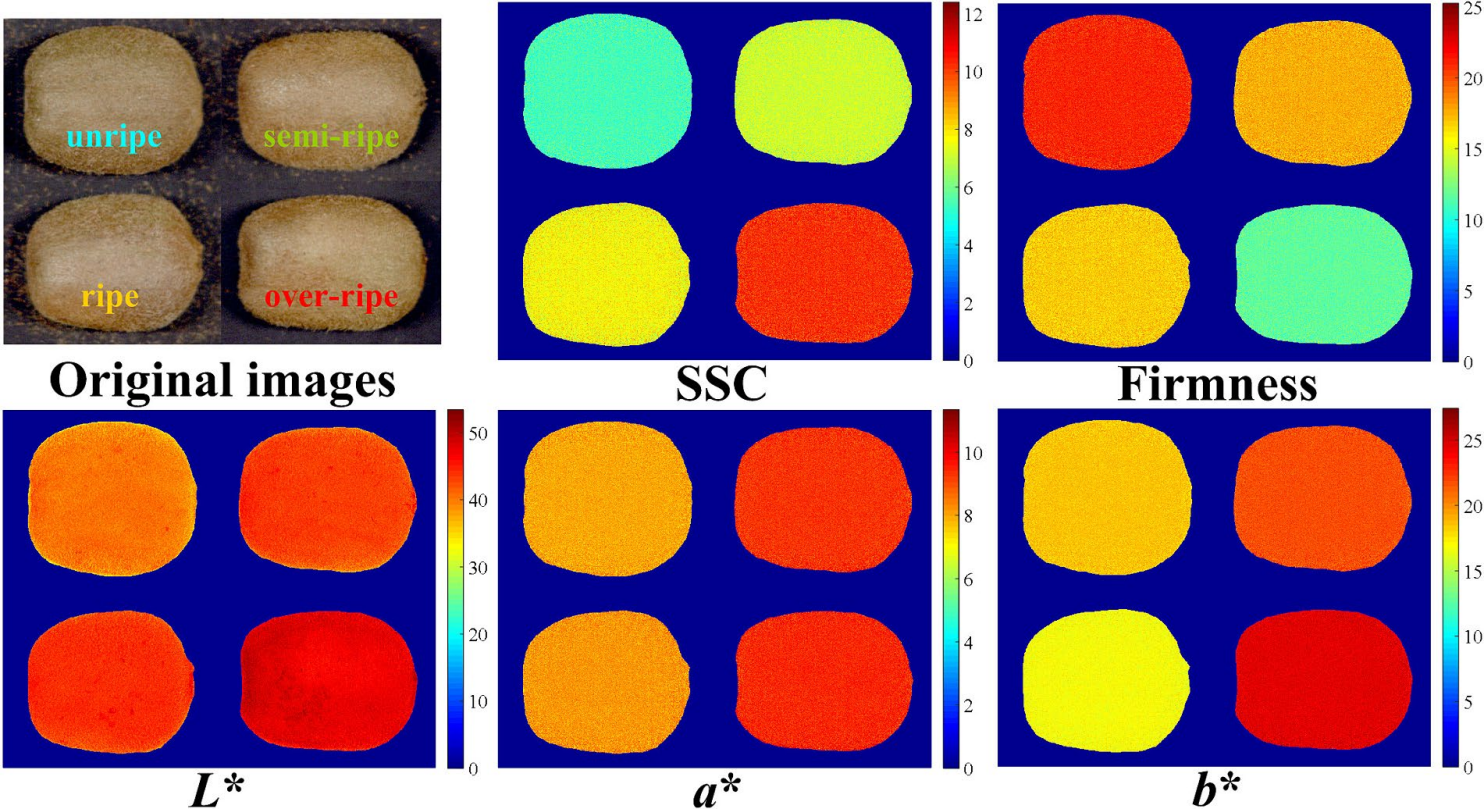
Visualization map of physicochemical indices of kiwifruits.

Hyperspectral imaging can acquire spectral information for each pixel in a test sample (23) and then use this information to generate visualization maps of reference values for physicochemical indices. In this manner, it is more intuitive to detect the reference values of the physicochemical indices of kiwifruit at different maturity stages. In this study, we inputted the spectral information of each pixel in the ROI of kiwifruit samples into the CARS-MLR model to predict the SSC, firmness, L^*^, a^*^, and b^*^ of each pixel of kiwifruits, and used the pseudo-color technique to visualize the distributions of these five indices at different maturity stages (24, 25). These distributions are shown in Figure 5. It can be observed from the visualized images that the color distributions of SSC and firmness of kiwifruits at different maturity stages differed significantly. In particular, the color distribution of SSC gradually became yellow (i.e., SSC gradually increased), whereas that of firmness gradually turned green (i.e., the firmness values gradually decreased) as kiwifruits gradually ripened, which was consistent with the results of the actual chemical experiments (26). The changes in the color distributions of L^*^, a^*^, and b^*^ of kiwifruits at different maturity stages were relatively small, and consistent with the statistical results of the physicochemical indices of kiwifruits described in Subsection 3.1.

## 4 Conclusion

A rapid detection model for the physicochemical indices (SSC, firmness, L^*^, a^*^, and b^*^) of kiwifruits at different maturity stages was developed using hyperspectral imaging and chemometrics, making it possible to visualize the distributions of the physicochemical indices of kiwifruits. Compared with the full-band detection model, the CARS algorithm was used to select 32, 18, 26, 29, and 32 feature wavelengths from 256 full-band wavelengths for the prediction of SSC, firmness, L^*^, a^*^, and b^*^, respectively, which improved the detection efficiency of the prediction model. Furthermore, the constructed CARS-MLR model exhibited the best performance in predicting the physicochemical indices. For this optimal CARS-MLR model, the prediction set *R*^2^_P_ was 0.89, 0.88, 0.81, 0.86, and 0.88, the RMSEP was 0.63, 1.09, 0.97, 0.47, and 0.97, and the RPD was 3.09, 2.90, 2.32, 2.74, and 2.91, respectively. The results indicated that the physicochemical indices of kiwifruits at different maturity stages can be rapidly and accurately detected. Based on the optimal detection model, the pseudo-color technique was used to visualize the distributions of the physicochemical indices of kiwifruits at different maturity stages, which made detecting these physicochemical indices more intuitive. The combination of hyperspectral imaging and chemometrics led to the rapid detection and distribution visualization of the physicochemical indices of kiwifruits, which laid a theoretical foundation for the development of nondestructive detection equipment for fruit quality and ripeness.

## Data availability statement

The original contributions presented in the study are included in the article/supplementary material, further inquiries can be directed to the corresponding author.

## Author contributions

QM: Conceptualization, Funding acquisition, Investigation, Project administration, Supervision, Writing – original draft, Writing – review & editing. TT: Data curation, Methodology, Resources, Software, Validation, Visualization, Writing – review & editing. SF: Data curation, Formal analysis, Methodology, Software, Validation, Visualization, Writing – review & editing. QW: Data curation, Formal analysis, Methodology, Software, Writing – review & editing. JS: Conceptualization, Funding acquisition, Investigation, Project administration, Supervision, Writing – original draft, Writing – review & editing.

## References

[1] Liu D Guo W Li Q Xie D. Relationship of the bulk optical properties in 950-1650 nm wavelength range with internal quality and microstructure of kiwifruit during maturation. Biosyst Eng. (2019) 184:45–54. doi: 10.1016/j.biosystemseng.2019.05.005

[2] Gao Q Wang P Niu T He D Wang M Yang H. Soluble solid content and firmness index assessment and maturity discrimination of *Malus micromalus Makino* based on near-infrared hyperspectral imaging. Food Chem. (2022) 370:131013. doi: 10.1016/j.foodchem.2021.131013, PMID: 34509150

[3] Lan W Jaillais B Renard C Leca A Chen S Bourvellec C. A method using near infrared hyperspectral imaging to highlight the internal quality of apple fruit slices. Postharvest Biol Tec. (2021) 175:111497–8. doi: 10.1016/j.postharvbio.2021.111497

[4] Chen H Qiao H Feng Q Xu L Cai K. Rapid detection of pomelo fruit quality using near-infrared hyperspectral imaging combined with chemometric methods. Front Bioeng Biotech. (2021) 8:616943. doi: 10.3389/fbioe.2020.616943, PMID: 33511105 PMC7835416

[5] Feng L Wu B Zhu S He Y Zhang C. Application of visible/infrared spectroscopy and hyperspectral imaging with machine learning techniques for identifying food varieties and geographical origins. Front Nutr. (2021) 8:680357. doi: 10.3389/fnut.2021.680357, PMID: 34222304 PMC8247466

[6] Garillos-Manliguez C Chiang J. Multimodal deep learning and visible-light and hyperspectral imaging for fruit maturity estimation. Sensors. (2021) 21:1–18. doi: 10.3390/s21041288, PMID: 33670232 PMC7916978

[7] Li B Cobo-Medina M Lecourt J Harrison N Harrison R Cross J. Application of hyperspectral imaging for nondestructive measurement of plum quality attributes. Postharvest Biol Tec. (2018) 141:8–15. doi: 10.1016/j.postharvbio.2018.03.008

[8] Riccioli C Pérez-Marín D Garrido-Varo A. Optimizing spatial data reduction in hyperspectral imaging for the prediction of quality parameters in intact oranges. Postharvest Biol Tec. (2021) 176:111504–7. doi: 10.1016/j.postharvbio.2021.111504

[9] Wang T Li G Dai C. Soluble solids content prediction for Korla fragrant pears using hyperspectral imaging and GsMIA. Infrared Phys Techn. (2022) 123:104119–8. doi: 10.1016/j.infrared.2022.104119

[10] Cheng J Sun D. Hyperspectral imaging as an effective tool for quality analysis and control of fish and other seafoods: current research and potential applications. Trends Food Sci Tech. (2014) 37:78–91. doi: 10.1016/j.tifs.2014.03.006

[11] Zhang H Zhan B Pan F Wei L. Determination of soluble solids content in oranges using visible and near infrared full transmittance hyperspectral imaging with comparative analysis of models. Postharvest Biol Tec. (2020) 163:111148–9. doi: 10.1016/j.postharvbio.2020.111148

[12] Fan S Huang W Guo Z Zhao C. Prediction of soluble solids content and firmness of pears using hyperspectral reflectance imaging. Food Anal Method. (2015) 8:1936–46. doi: 10.1007/s12161-014-0079-1

[13] Xie C Chu B He Y. Prediction of banana color and firmness using a novel wavelengths selection method of hyperspectral imaging. Food Chem. (2018) 245:132–40. doi: 10.1016/j.foodchem.2017.10.079, PMID: 29287354

[14] Su Z Zhang C Yan T Zhu J Zeng Y Lu X. Application of hyperspectral imaging for maturity and soluble solids content determination of strawberry with deep learning approaches. Front Plant Sci. (2021) 12:736334. doi: 10.3389/fpls.2021.736334, PMID: 34567050 PMC8462090

[15] Wang F Zhao C Yang H Jiang H Li L Yang G. Non-destructive and in-site estimation of apple quality and maturity by hyperspectral imaging. Comput Electron Agr. (2022) 195:106843. doi: 10.1016/j.compag.2022.106843

[16] Meng Q Shang J Huang R Zhang Y. Determination of soluble solids content and firmness in plum using hyperspectral imaging and chemometric algorithms. J Food Process Eng. (2021) 44:1–9. doi: 10.1111/jfpe.13597

[17] Tang N Sun J Yao K Zhou X Tian Y Nirere A. Identification of *Lycium barbarum* varieties based on hyperspectral imaging technique and competitive adaptive reweighted sampling-whale optimization algorithm-support vector machine. J Food Process Eng. (2021) 44:e13603. doi: 10.1111/jfpe.13603

[18] Ye S Wang D Min S. Successive projections algorithm combined with uninformative variable elimination for spectral variable selection. Chemomet Intell Lab. (2008) 91:194–9. doi: 10.1016/j.chemolab.2007.11.005

[19] Nicolaї B Beullens K Bobelyn E Peirs A Saeys W Theron K. Nondestructive measurement of fruit and vegetable quality by means of NIR spectroscopy: a review. Postharvest Biol Tec. (2007) 46:99–118. doi: 10.1016/j.postharvbio.2007.06.024

[20] Galvào R Araujo M José G Pontes M Silva E Saldanha T. A method for calibration and validation subset partitioning. Talanta. (2005) 67:736–40. doi: 10.1016/j.talanta.2005.03.025, PMID: 18970233

[21] Ding G Li B Han Y Liu A Zhang J Peng J. A rapid integrated bioactivity evaluation system based on near-infrared spectroscopy for quality control of *Flos Chrysanthemi*. J Pharmaceut Biomed. (2016) 131:391–9. doi: 10.1016/j.jpba.2016.09.008, PMID: 27643861

[22] Xiao Q Tang W Zhang C Zhou L Feng L Shen J. Spectral preprocessing combined with deep transfer learning to evaluate chlorophyll content in cotton leaves. Plant Phenomics. (2022) 2022:127–41. doi: 10.34133/2022/9813841, PMID: 36158530 PMC9489230

[23] Wang B He J Zhang S Li L. Nondestructive prediction and visualization of total flavonoids content in Cerasus Humilis fruit during storage periods based on hyperspectral imaging technique. J Food Process Eng. (2021) 44:e13807. doi: 10.1111/jfpe.13807

[24] Wang F Wang C Song S Xie S Kang F. Study on starch content detection and visualization of potato based on hyperspectral imaging. Food Sci Nutr. (2021) 9:4420–30. doi: 10.1002/fsn3.2415, PMID: 34401090 PMC8358368

[25] Song K Wang S Yang D Shi T. Combination of spectral and image information from hyperspectral imaging for the prediction and visualization of the total volatile basic nitrogen content in cooked beef. J Food Meas Charact. (2021) 15:4006–20. doi: 10.1007/s11694-021-00983-x

[26] Wang H Wang J Mujumdar A Jin X Liu Z Zhang Y. Effects of postharvest ripening on physicochemical properties, microstructure, cell wall polysaccharides contents (pectin, hemicellulose, cellulose) and nanostructure of kiwifruit (*Actinidia deliciosa*). Food Hydrocolloid. (2021) 118:106808. doi: 10.1016/j.foodhyd.2021.106808

